# A one-dimensional Ag^I^ coordination polymer: *catena*-poly[[[[*N*′-(4-cyano­benzyl­idene)nicotinohydrazide]silver(I)]-μ-*N*′-(4-cyano­benzyl­idene)nicotino­hydrazide] trifluoro­methane­sulfonate]

**DOI:** 10.1107/S1600536808034685

**Published:** 2008-10-31

**Authors:** Cao-Yuan Niu, Xin-Sheng Wan, Xiao-Wei Gui, Zhan-Guo Cao, Chun-Hong Kou

**Affiliations:** a College of Sciences, Henan Agricultural University, Zhengzhou 450002, People’s Republic of China

## Abstract

In the title compound, {[Ag(C_14_H_10_N_4_O)_2_]CF_3_SO_3_}_*n*_, the unique Ag^I^ ion is coordinated by two N atoms from two pyridine rings of two independent *N*′-(4-cyano­benzyl­idene)nicotinohydrazide ligands and one N atom of a carbonitrile group of a symmetry-related *N*′-(4-cyano­benzyl­idene)nicotino­hydrazide ligand, forming a distorted T-shaped coordination environment. One of the independent ligands acts as a bridge connecting Ag^I^ ions, forming chains along the *a* axis. In the crystal structure, two neighbouring anti­parallel chains are connected through N—H⋯O hydrogen bonds. In addition, there are relatively short Ag⋯O contacts of 2.723 (3) Å, which connect the chains into a three-dimensional structure.

## Related literature

For a related structure, see: Niu *et al.* (2007 ▶).
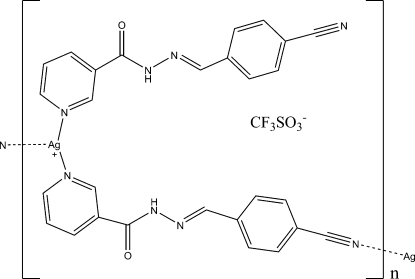


## Experimental

### 

#### Crystal data

[Ag(C_14_H_10_N_4_O)_2_]CF_3_SO_3_
*M* *_r_* = 757.46
Monoclinic, 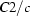

*a* = 24.966 (2) Å
*b* = 13.9529 (13) Å
*c* = 17.6976 (16) Å
β = 98.437 (2)°
*V* = 6098.3 (10) Å^3^
*Z* = 8
Mo *K*α radiation
μ = 0.80 mm^−1^
*T* = 173 (2) K
0.51 × 0.32 × 0.27 mm

#### Data collection

Siemens SMART CCD diffractometer
Absorption correction: multi-scan (*SADABS*; Sheldrick, 1996 ▶) *T*_min_ = 0.685, *T*_max_ = 0.813
19396 measured reflections
6990 independent reflections
5059 reflections with *I* > 2σ(*I*)
*R*_int_ = 0.030

#### Refinement

*R*[*F* ^2^ > 2σ(*F* ^2^)] = 0.049
*wR*(*F* ^2^) = 0.148
*S* = 1.03
6990 reflections
432 parameters
22 restraints
H atoms treated by a mixture of independent and constrained refinement
Δρ_max_ = 1.47 e Å^−3^
Δρ_min_ = −0.79 e Å^−3^

### 

Data collection: *SMART* (Siemens, 1996 ▶); cell refinement: *SAINT* (Siemens, 1996 ▶); data reduction: *SAINT*; program(s) used to solve structure: *SHELXS97* (Sheldrick, 2008 ▶); program(s) used to refine structure: *SHELXL97* (Sheldrick, 2008 ▶); molecular graphics: *DIAMOND* (Brandenburg, 2005 ▶); software used to prepare material for publication: *SHELXTL* (Sheldrick, 2008 ▶).

## Supplementary Material

Crystal structure: contains datablocks I, global. DOI: 10.1107/S1600536808034685/lh2709sup1.cif

Structure factors: contains datablocks I. DOI: 10.1107/S1600536808034685/lh2709Isup2.hkl

Additional supplementary materials:  crystallographic information; 3D view; checkCIF report

## Figures and Tables

**Table:** 

Ag1—N1	2.190 (3)
Ag1—N2	2.207 (3)
Ag1—N8^i^	2.518 (3)

**Table:** 

N1—Ag1—N2	158.96 (11)
N1—Ag1—N8^i^	100.66 (12)
N2—Ag1—N8^i^	96.76 (12)

Symmetry code: (i) 
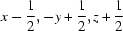
.

**Table 2:** Hydrogen-bond geometry (Å, °)

*D*—H⋯*A*	*D*—H	H⋯*A*	*D*⋯*A*	*D*—H⋯*A*
N3—H29⋯O2^ii^	0.869 (14)	2.18 (2)	2.999 (4)	156 (4)
N6—H28⋯O3	0.852 (18)	2.07 (2)	2.911 (5)	171 (3)

Symmetry code: (ii) 
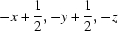
.

**Fig. 1.**
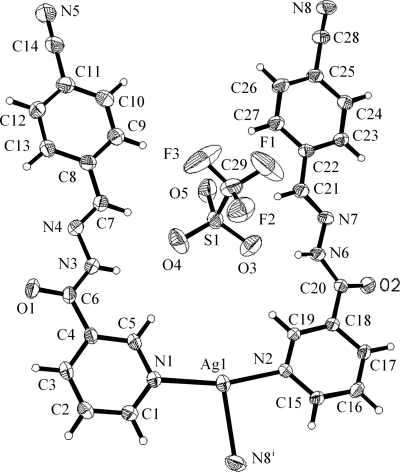
A view of the AgI coordination environment in the polymeric structure of (I), showing the atom-labelling scheme. Displacement ellipsoids are drawn at the 30% probability level and H atoms are shown as small spheres of arbitrary radii. [Symmetry codes: (i) x - 1/2, -y + 1/2, z + 1/2.]

**Fig. 2.**
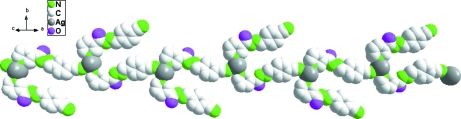
A space-filling diagram showing the one-dimensional chain. All counteranions and H atoms have been omitted for clarity.

**Fig. 3.**
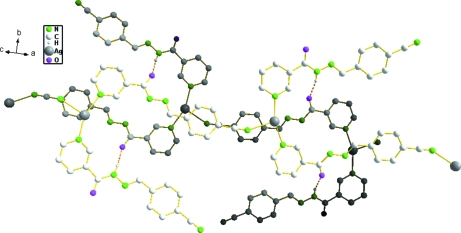
A diagram showing the intermolecular hydrogen bonds indicated by dashed lines. All counteranions and H atoms not involved in hydrogen bonds have been omitted for clarity.

**Fig. 4.**
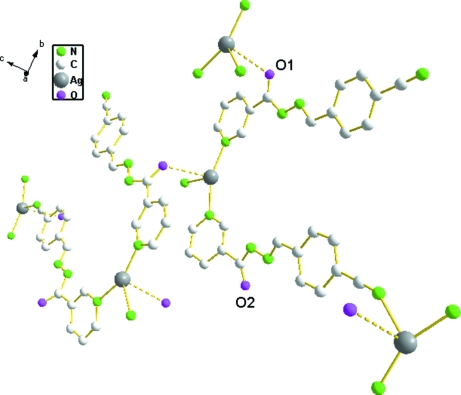
A diagram showing the intermolecular Ag···O interactions indicated by dashed lines. All counteranions and H atoms have been omitted for clarity.

## supplementary crystallographic information

### Comment

In the title compound, (I), the unique Ag^I^ ion is coordinated by two nitrogen atoms from two pyridyl rings of two different ligands (N1, N2) and one nitrogen atom from one carbonitrile group of another ligand [N8^i^. symmetry code: (i) *x* - 1/2, -*y* + 1/2, *z* + 1/2,] forming a slightly distorted T-shaped coordination enviroment (Fig. 1). The N1—Ag1—N2 bond angle is 158.96 (11), indicating these three atoms are not exactly linear. Thus, the N1—Ag1—N8^i^ and N2—Ag1—N8^i^ bond angles are larger than 90°. The N—Ag bond distances involving the pyridine rings are in the range of 2.190 (3)–2.207 (3) Å, which are smaller than N—Ag bond distance involving the carbonitrile group, 2.518 (3) Å. This probably indicates that nitrogen atoms of carbonitrile groups possess a weaker coordinating ability with silver than the nitrogen atoms of the pyridine rings in one ligand. In the crystal structure, half of the 4-cyanobenzylidene nicotinohydrazide molecules act as bridging ligands, the other half coordinating only in the monodentate mode. Differences in bond distances between N_pyridine_—Ag and N_carbonitrile_—Ag bonds can also be found in {[Ag_2_(1,6-Dihydro-2-methyl-6-oxo-(3,4'-bipyridine)-5-carbonitrile)_3_] _2_(CH_3_OH)_3_(PF_6_)_4_}*_n_* (Niu *et al.*, 2007), where the N_carbonitrile_—Ag bond distance of 2.529 (3) Å (similar to that in the title compound), is larger than the N_pyridine_—Ag bond distance of 2.151 (3) Å.

The ligands acting as µ_2_-bridging ligands coordinate through pyridine and carbonitrile nitrogen atoms. Each of these bridging ligands connects two silver atoms together by one pyridine nitrogen atom N1 and one carbonitrile nitrogen atom N8^i^ to form a one-dimensional chain along the *a* axis. The separation of two neighbouring silver atoms in one chain is ca. 16 Å, which means 4-cyanobenzylidene nicotinohydrazide acts as a moderately long bridging ligand. Interestingly, the other half of all ligands act only as monodentate terminal ligands and are coordinated to silver atoms in chains only through pyridine nitrogen atoms with the carbonitrile nitrogen atoms remaining uncoordinated. Two terminal ligands connecting to two adjacent silver atoms in one chain are located in opposite directions away from the chain. Thus, these chains possess a unusual 'saw-like' structure with the terminal ligands acting like 'saw-teeth' (Fig. 2).

In the crystal structure, the CF_3_SO_3_^-^ counteranions are connected the ligands of chains by N—H···O hydrogen bonds (Table 2). In addition, there are also N—H···O hydrogen bondings between two neighbouring antiparallel chains (Fig. 3). Furthermore, there are weak Ag···O interactions between one oxygen atom [O1] of the terminal ligand in one chain and one silver atom in the neighbouring chain with the Ag···O separation of 2.8760 (21) Å (Fig. 4). These noncovalent interactions have large contributions to the supramolecular three-dimensional framework of the compound.

### Experimental

A solution of AgCF_3_SO_3_ (0.026 g, 0.1 mmol) in CH_3_OH (10 ml) was carefully layered on a CH_3_OH/CHCl_3_ solution (5 ml/10 ml) of 4-Cyanobenzylidene nicotinohydrazide (0.025 g, 0.1 mmol) in a straight glass tube. About ten days later, colourless single crystals suitable for X-ray analysis were obtained (yield about 35%). Elementary analysis, calculated for C_29_H_20_AgN_8_O_5_F_3_S: C, 45.98, H, 2.66, N, 14.79%; found: C, 46.07, H, 2.53, N, 14.70%. One very strong bonds at 1262 cm^-1^ in the IR spectra were assigned to CF_3_SO_3_^-^.

### Refinement

C-bound H atoms were placed in calculated positions and refined using a riding model [C—H = 0.95 Å and *U*_iso_(H) = 1.2*U*_eq_(C)]. The N-bound H atoms were first introduced in calculated positions, and then thier positions and displacement parameters were refined with the N—H bond distance to 0.88 (2) Å, the distances of H29 and N4, H29 and C6 to 1.93 (2) and 1.96 (2) Å, respectively. The final difference Fourier map had a highest peak at 0.90 Å from atom Ag1 and a deepest hole at 0.76 Å from atom Ag1, but were otherwise featureless.

### Crystal data

**Table:**

[Ag(C_14_H_10_N_4_O)_2_]CF_3_SO_3_	*F*(000) = 3040
*M**_r_* = 757.46	*D*_x_ = 1.650 Mg m^−^^3^
Monoclinic, *C*2/*c*	Mo *K*α radiation, λ = 0.71073 Å
Hall symbol: -C 2yc	Cell parameters from 5153 reflections
*a* = 24.966 (2) Å	θ = 2.1–27.5°
*b* = 13.9529 (13) Å	µ = 0.80 mm^−^^1^
*c* = 17.6976 (16) Å	*T* = 173 K
β = 98.437 (2)°	Prism, colourless
*V* = 6098.3 (10) Å^3^	0.51 × 0.32 × 0.27 mm
*Z* = 8	

### Data collection

**Table:**

Siemens SMART CCD diffractometer	6990 independent reflections
Radiation source: fine-focus sealed tube	5059 reflections with *I* > 2σ(*I*)
graphite	*R*_int_ = 0.031
φ and ω scans	θ_max_ = 27.5°, θ_min_ = 2.1°
Absorption correction: multi-scan (SADABS; Sheldrick, 1996)	*h* = −28→32
*T*_min_ = 0.685, *T*_max_ = 0.813	*k* = −18→17
19396 measured reflections	*l* = −22→15

### Refinement

**Table:**

Refinement on *F*^2^	Primary atom site location: structure-invariant direct methods
Least-squares matrix: full	Secondary atom site location: difference Fourier map
*R*[*F*^2^ > 2σ(*F*^2^)] = 0.049	Hydrogen site location: inferred from neighbouring sites
*wR*(*F*^2^) = 0.148	H atoms treated by a mixture of independent and constrained refinement
*S* = 1.03	*w* = 1/[σ^2^(*F*_o_^2^) + (0.0766*P*)^2^ + 8.964*P*] where *P* = (*F*_o_^2^ + 2*F*_c_^2^)/3
6990 reflections	(Δ/σ)_max_ < 0.001
432 parameters	Δρ_max_ = 1.47 e Å^−^^3^
22 restraints	Δρ_min_ = −0.79 e Å^−^^3^

### Special details

**Table:**

Geometry. All e.s.d.'s (except the e.s.d. in the dihedral angle between two l.s. planes) are estimated using the full covariance matrix. The cell e.s.d.'s are taken into account individually in the estimation of e.s.d.'s in distances, angles and torsion angles; correlations between e.s.d.'s in cell parameters are only used when they are defined by crystal symmetry. An approximate (isotropic) treatment of cell e.s.d.'s is used for estimating e.s.d.'s involving l.s. planes.
Refinement. Refinement of *F*^2^ against ALL reflections. The weighted *R*-factor *wR* and goodness of fit *S* are based on *F*^2^, conventional *R*-factors *R* are based on *F*, with *F* set to zero for negative *F*^2^. The threshold expression of *F*^2^ > σ(*F*^2^) is used only for calculating *R*-factors(gt) *etc*. and is not relevant to the choice of reflections for refinement. *R*-factors based on *F*^2^ are statistically about twice as large as those based on *F*, and *R*- factors based on ALL data will be even larger.

### Fractional atomic coordinates and isotropic or equivalent isotropic displacement parameters (Å^2^)

**Table:**

	*x*	*y*	*z*	*U*_iso_*/*U*_eq_	
Ag1	0.177461 (13)	0.31617 (2)	0.21259 (2)	0.05965 (14)	
N1	0.16489 (13)	0.4710 (2)	0.22045 (18)	0.0481 (7)	
N2	0.19741 (12)	0.17730 (19)	0.16389 (17)	0.0421 (6)	
N3	0.25625 (13)	0.6644 (2)	0.10836 (19)	0.0488 (7)	
N4	0.29258 (12)	0.7215 (2)	0.07857 (19)	0.0497 (7)	
N5	0.5169 (2)	0.9140 (4)	−0.1204 (3)	0.1027 (17)	
N6	0.32032 (13)	0.1492 (2)	0.02428 (18)	0.0480 (7)	
N7	0.35916 (12)	0.1432 (2)	−0.02381 (18)	0.0491 (7)	
N8	0.60476 (14)	0.2456 (3)	−0.2154 (2)	0.0662 (10)	
S1	0.35594 (5)	0.41766 (7)	0.11529 (6)	0.0575 (3)	
O1	0.23222 (12)	0.78306 (19)	0.18438 (17)	0.0603 (7)	
O2	0.28597 (11)	0.0026 (2)	−0.01063 (16)	0.0589 (7)	
O3	0.33416 (17)	0.3230 (2)	0.1160 (2)	0.0852 (11)	
O4	0.32654 (16)	0.4890 (3)	0.1495 (2)	0.0886 (11)	
O5	0.37379 (17)	0.4448 (2)	0.04515 (19)	0.0852 (11)	
F1	0.4489 (2)	0.3413 (5)	0.1600 (3)	0.180 (2)	
F2	0.40950 (15)	0.3851 (3)	0.2507 (2)	0.1073 (11)	
F3	0.4445 (2)	0.4874 (4)	0.1883 (3)	0.180 (2)	
C1	0.12856 (16)	0.5082 (3)	0.2610 (2)	0.0548 (10)	
H1	0.1059	0.4658	0.2839	0.066*	
C2	0.12275 (17)	0.6049 (3)	0.2707 (3)	0.0611 (11)	
H2	0.0966	0.6289	0.2998	0.073*	
C3	0.15543 (18)	0.6664 (3)	0.2376 (3)	0.0562 (10)	
H3	0.1524	0.7336	0.2444	0.067*	
C4	0.19279 (14)	0.6307 (2)	0.19433 (19)	0.0410 (7)	
C5	0.19597 (15)	0.5322 (3)	0.1878 (2)	0.0452 (8)	
H5	0.2216	0.5065	0.1585	0.054*	
C6	0.22824 (15)	0.6994 (2)	0.1618 (2)	0.0437 (8)	
C7	0.32028 (16)	0.6787 (3)	0.0335 (2)	0.0497 (9)	
H7	0.3143	0.6126	0.0226	0.060*	
C8	0.36060 (15)	0.7304 (3)	−0.0009 (2)	0.0492 (9)	
C9	0.39393 (18)	0.6808 (3)	−0.0440 (3)	0.0629 (11)	
H9	0.3893	0.6137	−0.0514	0.075*	
C10	0.43334 (18)	0.7280 (4)	−0.0756 (3)	0.0657 (11)	
H10	0.4556	0.6936	−0.1054	0.079*	
C11	0.44089 (18)	0.8263 (3)	−0.0643 (3)	0.0610 (11)	
C12	0.40796 (18)	0.8751 (3)	−0.0220 (3)	0.0624 (11)	
H12	0.4131	0.9420	−0.0139	0.075*	
C13	0.36835 (17)	0.8298 (3)	0.0086 (2)	0.0549 (10)	
H13	0.3455	0.8654	0.0367	0.066*	
C14	0.4834 (2)	0.8750 (4)	−0.0962 (3)	0.0731 (13)	
C15	0.18030 (14)	0.0956 (3)	0.1923 (2)	0.0434 (8)	
H15	0.1556	0.0994	0.2283	0.052*	
C16	0.19682 (16)	0.0068 (3)	0.1717 (2)	0.0496 (9)	
H16	0.1842	−0.0494	0.1936	0.060*	
C17	0.23172 (15)	0.0003 (2)	0.1192 (2)	0.0440 (8)	
H17	0.2436	−0.0606	0.1042	0.053*	
C18	0.24964 (13)	0.0833 (2)	0.08797 (19)	0.0390 (7)	
C19	0.23178 (14)	0.1700 (2)	0.1127 (2)	0.0408 (7)	
H19	0.2445	0.2273	0.0924	0.049*	
C20	0.28677 (14)	0.0743 (2)	0.0289 (2)	0.0421 (8)	
C21	0.38690 (16)	0.2191 (3)	−0.0263 (2)	0.0513 (9)	
H21	0.3784	0.2739	0.0016	0.062*	
C22	0.43167 (14)	0.2241 (3)	−0.0711 (2)	0.0470 (8)	
C23	0.45139 (15)	0.1431 (3)	−0.1041 (2)	0.0496 (9)	
H23	0.4343	0.0828	−0.1004	0.059*	
C24	0.49545 (16)	0.1502 (3)	−0.1419 (2)	0.0516 (9)	
H24	0.5091	0.0949	−0.1639	0.062*	
C25	0.52007 (14)	0.2387 (3)	−0.1478 (2)	0.0481 (8)	
C26	0.50079 (18)	0.3193 (3)	−0.1168 (3)	0.0615 (11)	
H26	0.5176	0.3796	−0.1218	0.074*	
C27	0.45664 (18)	0.3123 (3)	−0.0780 (3)	0.0590 (11)	
H27	0.4433	0.3678	−0.0559	0.071*	
C28	0.56767 (15)	0.2436 (3)	−0.1854 (2)	0.0524 (9)	
C29	0.4169 (2)	0.4087 (4)	0.1813 (4)	0.0870 (16)	
H28	0.3208 (14)	0.1991 (18)	0.0520 (17)	0.034 (9)*	
H29	0.2522 (16)	0.6077 (15)	0.088 (2)	0.077 (15)*	

### Atomic displacement parameters (Å^2^)

**Table:**

	*U*^11^	*U*^22^	*U*^33^	*U*^12^	*U*^13^	*U*^23^
Ag1	0.0648 (2)	0.03572 (18)	0.0846 (3)	0.00461 (12)	0.03151 (17)	−0.00623 (14)
N1	0.0518 (17)	0.0365 (16)	0.0593 (18)	0.0005 (13)	0.0199 (14)	−0.0055 (13)
N2	0.0429 (15)	0.0342 (15)	0.0533 (17)	0.0026 (12)	0.0204 (13)	0.0006 (12)
N3	0.0516 (18)	0.0351 (17)	0.062 (2)	−0.0037 (13)	0.0157 (15)	−0.0009 (14)
N4	0.0453 (17)	0.0426 (17)	0.0613 (19)	−0.0032 (13)	0.0087 (14)	0.0035 (15)
N5	0.097 (3)	0.080 (3)	0.145 (5)	0.001 (3)	0.067 (3)	0.018 (3)
N6	0.0516 (18)	0.0436 (17)	0.0556 (18)	−0.0025 (14)	0.0308 (14)	−0.0074 (15)
N7	0.0481 (17)	0.0516 (18)	0.0530 (17)	0.0022 (14)	0.0257 (14)	0.0003 (15)
N8	0.055 (2)	0.083 (3)	0.067 (2)	−0.0100 (19)	0.0293 (17)	−0.003 (2)
S1	0.0696 (6)	0.0462 (5)	0.0620 (6)	0.0057 (5)	0.0276 (5)	−0.0020 (5)
O1	0.0777 (19)	0.0316 (13)	0.0750 (19)	−0.0016 (13)	0.0223 (15)	−0.0033 (13)
O2	0.0707 (18)	0.0435 (15)	0.0692 (18)	−0.0031 (13)	0.0324 (14)	−0.0152 (13)
O3	0.116 (3)	0.066 (2)	0.081 (2)	−0.0313 (19)	0.039 (2)	−0.0132 (17)
O4	0.107 (3)	0.085 (3)	0.078 (2)	0.038 (2)	0.029 (2)	−0.0101 (19)
O5	0.129 (3)	0.063 (2)	0.073 (2)	0.010 (2)	0.048 (2)	0.0106 (17)
F1	0.107 (3)	0.242 (6)	0.198 (5)	0.086 (3)	0.042 (3)	0.023 (4)
F2	0.105 (2)	0.118 (3)	0.094 (2)	−0.018 (2)	0.0007 (19)	0.033 (2)
F3	0.157 (4)	0.179 (5)	0.188 (4)	−0.111 (4)	−0.028 (3)	0.067 (4)
C1	0.051 (2)	0.047 (2)	0.072 (3)	−0.0022 (17)	0.0272 (19)	−0.0071 (19)
C2	0.061 (2)	0.050 (2)	0.080 (3)	0.0048 (19)	0.034 (2)	−0.014 (2)
C3	0.063 (2)	0.038 (2)	0.073 (3)	0.0095 (17)	0.024 (2)	−0.0100 (18)
C4	0.0428 (18)	0.0347 (18)	0.0459 (18)	0.0056 (14)	0.0082 (14)	−0.0045 (14)
C5	0.0493 (19)	0.0372 (18)	0.052 (2)	0.0074 (15)	0.0164 (16)	−0.0047 (15)
C6	0.0469 (19)	0.0330 (18)	0.051 (2)	0.0038 (14)	0.0069 (16)	−0.0007 (14)
C7	0.048 (2)	0.043 (2)	0.058 (2)	0.0004 (16)	0.0065 (17)	0.0016 (17)
C8	0.0431 (19)	0.053 (2)	0.051 (2)	−0.0008 (16)	0.0067 (16)	0.0048 (17)
C9	0.060 (3)	0.059 (3)	0.073 (3)	−0.001 (2)	0.020 (2)	−0.010 (2)
C10	0.061 (3)	0.072 (3)	0.069 (3)	0.004 (2)	0.027 (2)	−0.005 (2)
C11	0.052 (2)	0.067 (3)	0.065 (3)	0.0033 (19)	0.012 (2)	0.012 (2)
C12	0.066 (3)	0.049 (2)	0.076 (3)	−0.0001 (19)	0.025 (2)	0.011 (2)
C13	0.056 (2)	0.047 (2)	0.064 (2)	0.0069 (17)	0.0162 (19)	0.0032 (18)
C14	0.065 (3)	0.070 (3)	0.091 (3)	0.006 (2)	0.032 (3)	0.012 (3)
C15	0.0481 (19)	0.0362 (18)	0.0500 (19)	−0.0016 (14)	0.0212 (16)	−0.0007 (15)
C16	0.063 (2)	0.0335 (18)	0.057 (2)	−0.0047 (16)	0.0237 (18)	0.0053 (16)
C17	0.054 (2)	0.0294 (16)	0.052 (2)	−0.0002 (14)	0.0169 (16)	−0.0006 (14)
C18	0.0390 (17)	0.0349 (17)	0.0456 (18)	0.0005 (13)	0.0141 (14)	−0.0002 (14)
C19	0.0425 (18)	0.0305 (17)	0.053 (2)	−0.0001 (13)	0.0196 (15)	0.0028 (14)
C20	0.0427 (18)	0.0383 (18)	0.0485 (19)	0.0036 (14)	0.0172 (15)	0.0025 (15)
C21	0.054 (2)	0.050 (2)	0.056 (2)	−0.0010 (17)	0.0284 (18)	−0.0037 (18)
C22	0.0435 (19)	0.051 (2)	0.050 (2)	−0.0004 (16)	0.0188 (16)	0.0042 (17)
C23	0.047 (2)	0.047 (2)	0.058 (2)	−0.0052 (16)	0.0204 (17)	0.0013 (18)
C24	0.052 (2)	0.051 (2)	0.056 (2)	0.0028 (17)	0.0228 (17)	0.0001 (18)
C25	0.0424 (19)	0.059 (2)	0.0463 (19)	−0.0018 (16)	0.0185 (15)	0.0042 (17)
C26	0.059 (2)	0.057 (3)	0.076 (3)	−0.0119 (19)	0.033 (2)	0.001 (2)
C27	0.060 (2)	0.050 (2)	0.074 (3)	−0.0063 (18)	0.034 (2)	−0.0062 (19)
C28	0.047 (2)	0.065 (3)	0.048 (2)	−0.0058 (18)	0.0171 (16)	−0.0006 (18)
C29	0.071 (3)	0.080 (4)	0.115 (4)	−0.009 (3)	0.032 (3)	0.026 (3)

### Geometric parameters (Å, °)

**Table:**

Ag1—N1	2.190 (3)	C7—C8	1.444 (5)
Ag1—N2	2.207 (3)	C7—H7	0.9500
Ag1—N8^i^	2.518 (3)	C8—C9	1.392 (6)
N1—C5	1.341 (5)	C8—C13	1.407 (6)
N1—C1	1.341 (5)	C9—C10	1.370 (6)
N2—C19	1.340 (4)	C9—H9	0.9500
N2—C15	1.340 (4)	C10—C11	1.396 (7)
N3—C6	1.348 (5)	C10—H10	0.9500
N3—N4	1.370 (4)	C11—C12	1.372 (6)
N3—H29	0.869 (14)	C11—C14	1.442 (6)
N4—C7	1.278 (5)	C12—C13	1.351 (6)
N5—C14	1.133 (6)	C12—H12	0.9500
N6—C20	1.348 (5)	C13—H13	0.9500
N6—N7	1.383 (4)	C15—C16	1.372 (5)
N6—H28	0.852 (18)	C15—H15	0.9500
N7—C21	1.270 (5)	C16—C17	1.367 (5)
N8—C28	1.134 (5)	C16—H16	0.9500
N8—Ag1^ii^	2.518 (3)	C17—C18	1.385 (5)
S1—O4	1.424 (3)	C17—H17	0.9500
S1—O5	1.430 (3)	C18—C19	1.382 (5)
S1—O3	1.429 (3)	C18—C20	1.501 (4)
S1—C29	1.782 (6)	C19—H19	0.9500
O1—C6	1.232 (4)	C21—C22	1.464 (5)
O2—C20	1.220 (4)	C21—H21	0.9500
F1—C29	1.324 (8)	C22—C23	1.395 (6)
F2—C29	1.311 (6)	C22—C27	1.392 (5)
F3—C29	1.292 (7)	C23—C24	1.372 (5)
C1—C2	1.370 (6)	C23—H23	0.9500
C1—H1	0.9500	C24—C25	1.390 (6)
C2—C3	1.374 (6)	C24—H24	0.9500
C2—H2	0.9500	C25—C26	1.369 (6)
C3—C4	1.383 (5)	C25—C28	1.446 (5)
C3—H3	0.9500	C26—C27	1.385 (6)
C4—C5	1.382 (5)	C26—H26	0.9500
C4—C6	1.478 (5)	C27—H27	0.9500
C5—H5	0.9500		
			
N1—Ag1—N2	158.96 (11)	C12—C11—C14	121.0 (4)
N1—Ag1—N8^i^	100.66 (12)	C10—C11—C14	119.9 (4)
N2—Ag1—N8^i^	96.76 (12)	C13—C12—C11	121.3 (4)
C5—N1—C1	117.6 (3)	C13—C12—H12	119.4
C5—N1—Ag1	120.3 (2)	C11—C12—H12	119.4
C1—N1—Ag1	122.1 (3)	C12—C13—C8	120.6 (4)
C19—N2—C15	117.4 (3)	C12—C13—H13	119.7
C19—N2—Ag1	122.3 (2)	C8—C13—H13	119.7
C15—N2—Ag1	119.8 (2)	N5—C14—C11	179.1 (6)
C6—N3—N4	120.0 (3)	N2—C15—C16	122.9 (3)
C6—N3—H29	125.3 (15)	N2—C15—H15	118.5
N4—N3—H29	114.6 (15)	C16—C15—H15	118.5
C7—N4—N3	114.7 (3)	C17—C16—C15	119.2 (3)
C20—N6—N7	119.3 (3)	C17—C16—H16	120.4
C20—N6—H28	124 (2)	C15—C16—H16	120.4
N7—N6—H28	117 (2)	C16—C17—C18	119.4 (3)
C21—N7—N6	114.0 (3)	C16—C17—H17	120.3
C28—N8—Ag1^ii^	158.1 (4)	C18—C17—H17	120.3
O4—S1—O5	115.5 (2)	C19—C18—C17	117.8 (3)
O4—S1—O3	115.0 (2)	C19—C18—C20	123.7 (3)
O5—S1—O3	114.8 (2)	C17—C18—C20	118.5 (3)
O4—S1—C29	102.4 (3)	N2—C19—C18	123.3 (3)
O5—S1—C29	104.0 (3)	N2—C19—H19	118.3
O3—S1—C29	102.7 (3)	C18—C19—H19	118.3
N1—C1—C2	122.8 (4)	O2—C20—N6	124.0 (3)
N1—C1—H1	118.6	O2—C20—C18	120.5 (3)
C2—C1—H1	118.6	N6—C20—C18	115.5 (3)
C1—C2—C3	118.7 (4)	N7—C21—C22	121.4 (4)
C1—C2—H2	120.6	N7—C21—H21	119.3
C3—C2—H2	120.6	C22—C21—H21	119.3
C2—C3—C4	120.1 (3)	C23—C22—C27	119.3 (3)
C2—C3—H3	119.9	C23—C22—C21	122.2 (4)
C4—C3—H3	119.9	C27—C22—C21	118.4 (4)
C3—C4—C5	117.1 (3)	C24—C23—C22	120.2 (4)
C3—C4—C6	118.2 (3)	C24—C23—H23	119.9
C5—C4—C6	124.6 (3)	C22—C23—H23	119.9
N1—C5—C4	123.6 (3)	C23—C24—C25	119.7 (4)
N1—C5—H5	118.2	C23—C24—H24	120.1
C4—C5—H5	118.2	C25—C24—H24	120.1
O1—C6—N3	123.1 (4)	C26—C25—C24	120.9 (3)
O1—C6—C4	120.8 (3)	C26—C25—C28	120.5 (4)
N3—C6—C4	116.1 (3)	C24—C25—C28	118.6 (4)
N4—C7—C8	120.3 (4)	C25—C26—C27	119.5 (4)
N4—C7—H7	119.9	C25—C26—H26	120.2
C8—C7—H7	119.9	C27—C26—H26	120.2
C9—C8—C13	118.2 (4)	C26—C27—C22	120.3 (4)
C9—C8—C7	119.5 (4)	C26—C27—H27	119.8
C13—C8—C7	122.3 (4)	C22—C27—H27	119.8
C10—C9—C8	120.5 (4)	N8—C28—C25	178.6 (5)
C10—C9—H9	119.7	F3—C29—F2	105.7 (6)
C8—C9—H9	119.7	F3—C29—F1	107.3 (6)
C9—C10—C11	120.2 (4)	F2—C29—F1	105.2 (5)
C9—C10—H10	119.9	F3—C29—S1	113.3 (4)
C11—C10—H10	119.9	F2—C29—S1	114.0 (4)
C12—C11—C10	119.1 (4)	F1—C29—S1	110.8 (5)
			
N2—Ag1—N1—C5	−34.0 (5)	C19—N2—C15—C16	0.6 (5)
N8^i^—Ag1—N1—C5	−179.4 (3)	Ag1—N2—C15—C16	−171.5 (3)
N2—Ag1—N1—C1	149.7 (3)	N2—C15—C16—C17	−0.9 (6)
N8^i^—Ag1—N1—C1	4.4 (3)	C15—C16—C17—C18	0.0 (6)
N1—Ag1—N2—C19	27.7 (5)	C16—C17—C18—C19	1.0 (5)
N8^i^—Ag1—N2—C19	173.4 (3)	C16—C17—C18—C20	−178.4 (3)
N1—Ag1—N2—C15	−160.7 (3)	C15—N2—C19—C18	0.6 (5)
N8^i^—Ag1—N2—C15	−14.9 (3)	Ag1—N2—C19—C18	172.4 (3)
C6—N3—N4—C7	174.1 (3)	C17—C18—C19—N2	−1.4 (5)
C20—N6—N7—C21	177.1 (4)	C20—C18—C19—N2	178.0 (3)
C5—N1—C1—C2	−0.9 (6)	N7—N6—C20—O2	−4.8 (6)
Ag1—N1—C1—C2	175.5 (3)	N7—N6—C20—C18	174.3 (3)
N1—C1—C2—C3	0.1 (7)	C19—C18—C20—O2	−153.1 (4)
C1—C2—C3—C4	1.0 (7)	C17—C18—C20—O2	26.3 (5)
C2—C3—C4—C5	−1.2 (6)	C19—C18—C20—N6	27.7 (5)
C2—C3—C4—C6	−178.1 (4)	C17—C18—C20—N6	−152.9 (3)
C1—N1—C5—C4	0.6 (6)	N6—N7—C21—C22	176.8 (3)
Ag1—N1—C5—C4	−175.8 (3)	N7—C21—C22—C23	−9.6 (6)
C3—C4—C5—N1	0.4 (6)	N7—C21—C22—C27	173.2 (4)
C6—C4—C5—N1	177.1 (3)	C27—C22—C23—C24	1.0 (6)
N4—N3—C6—O1	2.4 (6)	C21—C22—C23—C24	−176.2 (4)
N4—N3—C6—C4	−176.0 (3)	C22—C23—C24—C25	−0.6 (6)
C3—C4—C6—O1	14.1 (5)	C23—C24—C25—C26	−0.4 (6)
C5—C4—C6—O1	−162.6 (4)	C23—C24—C25—C28	177.7 (4)
C3—C4—C6—N3	−167.4 (4)	C24—C25—C26—C27	1.0 (7)
C5—C4—C6—N3	15.9 (5)	C28—C25—C26—C27	−177.0 (4)
N3—N4—C7—C8	−179.1 (3)	C25—C26—C27—C22	−0.6 (7)
N4—C7—C8—C9	173.9 (4)	C23—C22—C27—C26	−0.4 (7)
N4—C7—C8—C13	−4.9 (6)	C21—C22—C27—C26	176.9 (4)
C13—C8—C9—C10	0.4 (6)	O4—S1—C29—F3	−62.1 (6)
C7—C8—C9—C10	−178.4 (4)	O5—S1—C29—F3	58.4 (6)
C8—C9—C10—C11	0.7 (7)	O3—S1—C29—F3	178.4 (5)
C9—C10—C11—C12	−0.8 (7)	O4—S1—C29—F2	58.8 (5)
C9—C10—C11—C14	178.4 (4)	O5—S1—C29—F2	179.4 (4)
C10—C11—C12—C13	−0.3 (7)	O3—S1—C29—F2	−60.7 (5)
C14—C11—C12—C13	−179.4 (4)	O4—S1—C29—F1	177.3 (4)
C11—C12—C13—C8	1.5 (7)	O5—S1—C29—F1	−62.2 (5)
C9—C8—C13—C12	−1.5 (6)	O3—S1—C29—F1	57.8 (5)
C7—C8—C13—C12	177.3 (4)		

Symmetry codes: (i) *x*−1/2, −*y*+1/2, *z*+1/2; (ii) *x*+1/2, −*y*+1/2, *z*−1/2.

### Hydrogen-bond geometry (Å, °)

**Table:**

*D*—H···*A*	*D*—H	H···*A*	*D*···*A*	*D*—H···*A*
N3—H29···O2^iii^	0.87 (1)	2.18 (2)	2.999 (4)	156 (4)
N6—H28···O3	0.85 (2)	2.07 (2)	2.911 (5)	171 (3)

Symmetry codes: (iii) −*x*+1/2, −*y*+1/2, −*z*.
